# Improving outcomes for asthma patients with allergic rhinitis: the MetaForum conferences

**DOI:** 10.1186/1471-2466-6-S1-S1

**Published:** 2006-11-30

**Authors:** Stephen T Holgate, David Price

**Affiliations:** 1 Infection, Inflammation and Repair AIR Division, Level F, South Block, MP810, Southampton General Hospital, Tremona Road, Southampton SO16 6YD, UK; 2 Department of General Practice and Primary Care, University of Aberdeen, Foresterhill Health Centre, Westburn Road, Aberdeen AB25 2AY, UK

## 

Asthma is one of the most common chronic diseases worldwide, affecting an estimated 300 million people around the globe [1]. The cause of one of every 250 deaths annually, asthma is also associated with high costs both economically and socially in terms of reduced quality of life and of missed work days and school days for patients and their families [1,2]. A large percentage of children and adults with asthma also have allergic rhinitis. The reported lifetime prevalence of allergic rhinitis among adults with asthma ranges from 50% to 100%, varying by study design and geographic locale [3]. While allergic rhinitis is not a life-threatening disease, its toll on quality of life, sleep, and daily functioning is well documented [4-7].

Asthma and allergic rhinitis are both inflammatory diseases of the airways. The similarities between allergic rhinitis and asthma in epidemiologic and pathophysiologic features suggest that allergic rhinitis and asthma represent manifestations of the same syndrome, the chronic allergic respiratory syndrome [8]. The Allergic Rhinitis Impact on Asthma (ARIA) report in 2001 [5] summarized the evidence supporting and describing the frequent clinical association between asthma and allergic rhinitis and the detrimental impact of allergic rhinitis on asthma. Citing the concept of 'one airway, one disease,' the ARIA report recommends that patients with persistent allergic rhinitis be screened for asthma and those patients with asthma be screened for allergic rhinitis, and that a combined strategy be used to treat both upper and lower airways. Many of the recommendations in ARIA relating to asthma management, however, were not reflected in subsequent clinical guidelines for asthma, including the Global Initiative for Asthma guidelines [9,10]. Moreover, asthma and allergic rhinitis often are not diagnosed in clinical practice [11].

Two international meetings were developed in response to the need to highlight the role of inflammation in asthma and the need for improved recognition of the relationship between asthma and allergic rhinitis. Both meetings were held under the auspices of the University of Southampton and the International Primary Care Respiratory Group, and were made possible through an educational grant by Merck & Co., Inc.

The first of these MetaForum conferences, held in London in April 2004, was entitled 'Improving Asthma Therapy through More Effective Control of Inflammation.' The second meeting, 'MetaForum: Improving Outcomes for Asthma Patients with Allergic Rhinitis,' took place in December 2004 in London and, like the first, brought together more than 40 leading experts in asthma and allergic rhinitis from 20 countries. These meetings included several presentations followed by an active discussion to reach consensus on the areas for action to improve outcomes for patients with asthma and allergic rhinitis. In the present supplement we include several of the major papers presented at these conferences, expanded and updated with more recent references.

## Competing interests

STH has received fees for lectures from Novartis and Merck Sharp & Dohme and is a consultant for Novartis, MRL, Almiral Prodesfarma, Rotta Pharm., Cambridge Antibody Technology, Amgen, Wyeth, UCB/Celltech, Avontec and Synairgen. DP has received honoraria for speaking at sponsored meetings from the following companies marketing respiratory products: 3M, Altana, AstraZeneca, Boehringer Ingelheim, GlaxoSmithKline, IVAX, Merck Sharp & Dohme, Novartis, Pfizer and Schering-Plough. DP has also received honoraria for advisory panels with 3M, Altana, AstraZeneca, Boehringer Ingelheim, GlaxoSmithKline, IVAX, MSD, Novartis, Pfizer and Schering-Plough. DP or his research team have received funding for research projects from 3M, Altana, AstraZeneca, Boehringer Ingelheim, GlaxoSmithKline, IVAX, Merck, Sharp & Dohme, Novartis, Pfizer, Schering-Plough, Viatris.

## References

[B1] Masoli M, Fabian D, Holt S, Beasley R (2004). The global burden of asthma: executive summary of the GINA Dissemination Committee report. Allergy.

[B2] Bousquet J, Bousquet PJ, Godard P, Daures JP (2005). The public health implications of asthma. Bull World Health Org.

[B3] Gaugris S, Sazonov-Kocevar V, Thomas M (2006). Burden of concomitant allergic rhinitis in adults with asthma. J Asthma.

[B4] Greiner AN (2006). Allergic rhinitis: impact of the disease and considerations for management. Med Clin North Am.

[B5] Bousquet J, Van Cauwenberge P, Khaltaev N (2001). Allergic rhinitis and its impact on asthma. J Allergy Clin Immunol.

[B6] Bousquet J, Neukirch F, Bousquet PJ, Gehano P, Klossek JM, Le Gal M, Allaf B (2006). Severity and impairment of allergic rhinitis in patients consulting in primary care. J Allergy Clin Immunol.

[B7] Laforest L, Bousquet J, Pietri G, Sazonov Kocevar V, Yin D, Pacheco Y, Van Ganse E (2005). Quality of life during pollen season in patients with seasonal allergic rhinitis with or without asthma. Int Arch Allergy Immunol.

[B8] Pawankar R (2006). Allergic rhinitis and asthma: are they manifestations of one syndrome?. Clin Exp Allergy.

[B9] National Institutes of Health, National Heart, Lung, Blood Institute (2002). Asthma management and prevention. Global initiative for asthma. A practical guide for public health officials and health care professionals. Based on the global strategy for asthma management and prevention NHLBI/WHO workshop report. Updated report 2002.

[B10] Global Initiative for Asthma 2005 Update: Workshop Report, Global Strategy for Asthma Management and Prevention. http://www.ginasthma.com/.

[B11] Nolte H, Nepper-Christensen S, Backer V (2006). Unawareness and undertreatment of asthma and allergic rhinitis in a general population. Respir Med.

